# Fault Detection and Isolation via the Interacting Multiple Model Approach Applied to Drive-By-Wire Vehicles

**DOI:** 10.3390/s18072332

**Published:** 2018-07-18

**Authors:** Vincent Judalet, Sébastien Glaser, Dominique Gruyer, Saïd Mammar

**Affiliations:** 1 LIVIC Laboratory, IFSTTAR, 25 Allée des Marronniers, 78000 Versailles, France; dominique.gruyer@ifsttar.fr; 2 Centre for Accident Research and Road Safety (CARRS-Q), Queensland University of Technology (QUT), Brisbane City, QLD 4000, Australia; sebastien.glaser@qut.edu.au; 3 IBISC Laboratory, University of Evry Val d’Essonne, 40 rue du Pelvoux, 91020 Evry, France; said.mammar@ibisc.univ-evry.fr

**Keywords:** Interacting Multiple Model, fault detection and isolation, drive-by-wire, extended Kalman filter

## Abstract

The place of driving assistance systems is currently increasing drastically for road vehicles. Paving the road to the fully autonomous vehicle, the drive-by-wire technology could improve the potential of the vehicle control. The implementation of these new embedded systems is still limited, mainly for reliability reasons, thus requiring the development of diagnostic mechanisms. In this paper, we investigate the detection and the identification of sensor and actuator faults for a drive-by-wire road vehicle. An Interacting Multiple Model approach is proposed, based on a non-linear vehicle dynamics observer. The adequacy of different probabilistic observers is discussed. The results, based on experimental vehicle signals, show a fast and robust identification of sensor faults while the actuator faults are more challenging.

## 1. Introduction

To enable advanced driver-assistance systems (ADAS) to operate more efficiently without disrupting the driver, decoupled control systems, known as drive-by-wire technologies, have been developed for the control of the steering or braking systems.

The drive-by-wire technology (DBW) consists in introducing electronic control systems between the driver interfaces (steering wheel and pedals) and the vehicle actuators (motor, brakes, and steering rack) in substitution for the conventional mechanical or hydraulic physical connections. According to the driver actions on both the steering wheel and the pedals, these systems automatically adapt and control the actuators to produce the desired vehicle behaviour.

The DBW systems present many advantages when compared to conventional systems, in terms of safety (via the active steering), comfort (configurable haptic feedback), vehicle design and costs (see [1]). However, by-wire systems pose significant challenges to manufacturers for their implementation. On the one hand, electrical systems are subject to failure in a less predictable and more frequent manner than mechanical systems. While the throttle-by-wire (or electronic throttle control) was successfully introduced to the market at the end of the 1990s, car manufacturers have faced many reliability and safety issues during the implementation in the market of other DBW systems, such as brake-by-wire (BBW) and steer-by-wire (SBW). For example, the first introduction of a SBW system by the manufacturer Infinity on the Q50 model led to a recall in 2013. This explains why the development of the DBW technology is still very limited.

In the aeronautic domain, the development of the fly-by-wire technology has been made possible by the use of the redundancy of sensors and actuators. This solution is however not compatible with cost-effectiveness constraints inherent to vehicles mass production, and the systems developed are generally limited to high-end models.

For a road vehicle, the steering and braking systems are independent, however they both take part in the control of the vehicle trajectory. We propose to take advantage of this diversity of control strategies to maintain a controllability (even though reduced) of the vehicle if a default occurs on one of these systems, as in [2]. For instance, if a fault occurs in the braking system, asymmetrical braking forces will disrupt the vehicle trajectory. In this case, the steering controller could automatically reject this perturbation. Similarly, if the steering system is faulty, differential braking could help to maintain the desired trajectory.

Such a reconfigurable control mechanism requires the real time diagnosis of a fault before its effect leads the vehicle to an uncontrollable state. As this fault may concern either the SBW or the BBW systems, both systems have to be supervised. Published research in the field of fault detection and isolation (FDI) applied to drive-by-wire vehicles treats separately the case of SBW [3,4,5,6,7,8,9,10] or BBW [3,11,12] system faults.

In this paper, we investigate a fast and reliable FDI scheme applied to a drive-by-wire vehicle. Both SBW and BBW systems fault are processed in a unique fault detection algorithm. To cope with the diversity of possible faults, the FDI algorithm relies on an Interacting Multiple Model (IMM) approach.

The considered DBW vehicle architecture is composed of different components:

- The human–machine interfaces (HMI) are typically the steering wheel and the pedals (brake and throttle). They collect the driver’s input and provide him with haptic feedback (wheel alignment torque for instance) and eventual incentive information.
- The sensor set corresponds to a standard vehicle equipped with an ESC (electronic stability control) system: wheel speed sensors, an inertial navigation system (INS), including accelerometers and gyrometers, a steering wheel angle sensor (SAS), pedals stroke and braking pressure sensors. Moreover, a sensor for the steering angle of the front wheels is required as it is subjected to differ from the steering wheel angle.
- The actuators include the brakes (electronic or electro-hydraulic) and the electronic steering system.
- The control unit supervises the system, i.e., estimates the vehicle dynamics, identifies eventual faults, and controls the actuators consequently.

Faults are subjected to occur on each of these systems. However, the faults occurring on the electronic control unit or on the HMI sensors (pedal stroke and SAS) are generally difficult to process without physical redundancies [13,14]. In this paper, we focus on the detection of abrupt partial and total faults on the following sensors and actuators:

- Sensor faults: X- and Y-accelerations, vehicle yaw rate, wheel turn rates and the steering angle of front wheels.
- Actuator faults: Steering actuator and brakes.

While the detection of multiple faults is compatible with the IMM approach, this subject is not addressed in this study. We consider the subsystems (sensors and actuators) are independent, so a fault is unlikely to affect two subsystems, and that a single failure will be detected before the occurrence of a second fault.

Many FDI approaches have been proposed in the literature (see [15,16,17,18,19]). On the one hand, data-based methods are based on the knowledge of a priori observation of the system. Quantitative approaches use artificial intelligence algorithms (neural networks and Bayesian frameworks) or the statistical processing of the data, while qualitative methods generally consist of expert systems. These approaches do not require any analytical or structural knowledge on the physical system.

On the other hand, model-based methods involve the physical knowledge of the monitored system. This knowledge leads to one or many models (analytical or graphical), which describes the system operating in normal state or under a degraded mode (for instance, see [20,21,22,23]).

Qualitative methods rely on the modelling of the input–output relationships in terms of quantitative functions. These methods are relatively easy to implement because they do not require information of the internal physical relations of the system, but are limited to commutative systems.

Quantitative model-based methods are based on the analytical modelling of the relations between the input and the output of the system, generally via a state analysis.

In the field of automotive control, the observation of the vehicle dynamics has been intensively investigated, and several evolution models are available. This explains why the literature on FDI schemes dedicated to SBW or BBW systems mainly rely on model-based methods [4,5,6,7,8,10,22,24].

Model-based FDI generally implies the generation of fault indicators, called residuals, obtained by comparing the predicted state of the system to the actual observed state. If the system is in the normal state, the residuals remain close to zero, else a fault is detected. The classification of the residuals eventually leads to the fault identification.

When an abrupt fault occurs on a system, its behavior may be radically altered and compromised. Thus, it is difficult to model this system both in the normal state and in the different degraded modes with a unique model. Multiple model (MM) approaches have been developed to cope with this issue (see [25,26]). They consist in implementing different observers in parallel; each observer corresponds to a specific system state (normal or degraded).

Originally, the observers were running independently, without any mutual interaction. This method is adequate when the model structure is not or slightly modified (for instance, parameter estimation problems), but if the models differ radically (which is generally the case in a FDI scheme), the transition from one state to another may be difficult to evaluate, thus jeopardizing the fault detection or increasing the detection time.

The Interacting Multiple Model (IMM) approach has been introduced to solve this problem (see [27,28,29,30,31]). The different system states are modelled in a Markov chain. Before each algorithm iteration, the estimates for each mode are updated according to the transition probabilities from one mode to another.

In the case of road vehicles, precise analytical models are already available in the literature. They are mainly based on the modelling of the forces between the tyre and the road to estimate the vehicle dynamics.

For this study, we implemented an IMM-based FDI approach to investigate the detection and the isolation of faults occurring on a DBW vehicle.

The paper is organized as follows: Section 2 introduces the IMM algorithm and its implementation for a FDI purpose. Section 3 presents the probabilistic observers, which have been considered for the IMM implementation. The non-linear vehicle model is detailed in Section 4, and finally the experimental validation tests are shown in Section 5.

## 2. The IMM Approach for the Fault Detection and Isolation

### 2.1. Principles of the IMM Estimation

In an IMM scheme, each system state is modelled in a separate model. To enable the interactions between the different modes, the transition probabilities from one mode to the others are considered. The interaction implies to correct the state estimates ${\hat{x}}_{i}$ for different modes towards the most likely estimate according to the transition probabilities $\pi_{ij}$ defined by:

(1) $$\pi_{ij}=p\left\{m_{j,k+1}|m_{i,k}\right\},\;\forall \left[m_{i},m_{j}\right]\in S^{2}$$

and

(2) $$\sum_{j=1}^{s}\pi_{ij}=1,\;\forall i\in \left[1,s\right]$$

where $S=\{m_{1},m_{2},\ldots ,m_{s}\}$ is the set of system modes, $\pi_{ij}$ is the transition probability from mode $m_{i}$ to mode $m_{j}$, and $m_{i,k}$ denotes that the system is in mode $m_{i}$ at time *k*.

The IMM estimator is a recursive algorithm. For each cycle, four steps are carried out:

1. First, the different estimates and their covariance matrices are mixed, according to the probabilities of activation $\mu_{i}$ of the model at the current time *k*. The predicted mode probability $\mu_{j}$, defined by:
  (3) $$\mu_{j,k+1|k}=\sum_{i}\pi_{ij}\mu_{i,k}$$
  enables computing the mixed probability $\mu_{i|j,k}$:
  (4) $$\mu_{i|j,k}=\frac{\pi_{ij}\mu_{i,k}}{\mu_{j,k+1|k}}$$
  The mixed estimates ${\hat{x}}_{j,k-1}^{0}$ are then defined by:
  (5) $${\hat{x}}_{j,k}^{0}=\sum_{i=1}^{s}\mu_{i|j,k}{\hat{x}}_{i,k}$$
  and their covariance matrices $P_{j,k-1}^{0}$ by:
  (6) $$P_{j,k}^{0}=\sum_{i=1}^{s}\left[P_{i,k}+\Delta {\hat{x}}_{i|j}\right]\mu_{i|j,k}$$
  where $\Delta {\hat{x}}_{i|j}$ is the model uncertainty:
  (7) $$\Delta {\hat{x}}_{i|j}=\left({\hat{x}}_{j,k}^{0}-{\hat{x}}_{i,k}\right){\left({\hat{x}}_{j,k}^{0}-{\hat{x}}_{i,k}\right)}^{t}$$
2. Second, a probabilistic filter is performed for each mode in parallel to obtain the updated estimates ${\hat{x}}_{j,k+1}$ and their covariance $P_{j,k+1}$. Starting from each mixed estimate ${\hat{x}}_{j,k}^{0}$, an evolution model is used to predict the next state vector, and then this prediction is corrected according to the last measured data. In Section 3, different probabilistic filters are investigated: the extended Kalman filter (EKF), the unscented Kalman filter (UKF), and the (first-order) divided differences filter (DD1).
3. The activation probability update is done with the computation of the likelihood $L_{j}$:
  (8) $$L_{j,k}=\frac{1}{{\left(2\pi \right)}^{d/2}\sqrt{\det\Lambda_{j,k}}}\exp\left[-\frac{1}{2}\nu_{j,k}^{T}\Lambda_{j,k}^{-1}\nu_{j,k}\right]$$
  where $\Lambda_{j,k}$ is the residual covariance matrix, as defined in Equation (17), and *d* is the dimension of the residual vector $\nu_{j,k}$. The updated probability for the mode *j* is obtained from the current likelihood and the past activation probability:
  (9) $$\mu_{j,k+1}=\frac{\mu_{j,k+1|k}L_{j,k+1}}{\sum_{i}\mu_{i,k+1|k}L_{i,k+1}}$$
4. Finally, the overall estimate ${\hat{x}}_{k+1}$ and an overall covariance matrix $P_{k+1}$ are computed by weighting the different estimates by their respective probabilities. This step is optional and is required only if an overall estimate is needed, for instance for control purpose.
  (10) $${\hat{x}}_{k+1}=\sum_{j}\mu_{j,k+1}{\hat{x}}_{j,k+1}$$
  (11) $$P_{k+1}=\sum_{j}\mu_{j,k+1}\left[P_{j,k+1}+\Delta {\hat{x}}_{j}\Delta {\hat{x}}_{j}^{t}\right]$$
  where
  (12) $$\Delta {\hat{x}}_{j}={\hat{x}}_{j,k+1}-{\hat{x}}_{k+1}$$

### 2.2. Dedicated Implementation for FDI Purpose

The first mode $m_{1}$, called *nominal* mode (NOM), corresponds to the normal (i.e., fault-free) state of the system. Next to the nominal mode, an additional *faulty* mode is implemented for each of the investigated faults.

A typical SBW vehicle is equipped with four wheel turn rate sensors, an inertial sensor (*x* and *y* accelerations and the vehicle yaw rate) and a steering angle sensor at the front wheels. Consequently, for the sensor faults detection, eight additional modes are proposed; their names are preceded by an “S” (for sensor):

- Four modes ($m_{2,3,4,5}$) (called S $\Omega_{FR}$, S $\Omega_{FL}$, S $\Omega_{RL}$ and S $\Omega_{RR}$) correspond to a fault on the wheel turn rate signals (respectively at the front right, front left, rear left and rear right wheels.
- Three modes ($m_{6,7,8}$) are for the inertial sensor faults (S A${}_{X}$ and S A${}_{Y}$ for the longitudinal and lateral accelerations, and S YR for the yaw rate).
- One mode ($m_{9}$) is for a fault on the front steering angle signal (S δ).

Likewise, five modes are introduced for the actuator fault detection (preceded by an “A” for actuator):

- One mode ($m_{10}$) is for a fault on the steering angle actuator (A δ).
- Four modes ($m_{11,12,13,14}$) (called A Br${}_{FR}$, A Br${}_{FL}$, A Br${}_{RL}$ and A Br${}_{RR}$) correspond to a fault on the braking system (respectively, at the front right, front left, rear left and rear right wheels).

In total, 14 modes have been considered.

No hypothesis is made on the presence of a fault at the start of the algorithm. Therefore, the different activation probabilities are initialized to the same value $\mu_{0}=1/n$ (where *n* is the number of modes).

In the fault-free state, the likelihood of the nominal mode should largely exceed those of the other (faulty) modes, thus the probability $\mu_{1}$ should rapidly rise to 100%.

When a fault occurs, the likelihood of all modes should be affected, except the mode corresponding to the affected sensor. The probability $\mu_{1}$ should then drop to 0 and the probability of the faulty mode should rise to 100%.

The detection and the isolation of the faults can consequently be carried out at the same time by comparing the probabilities of activation to a threshold $\mu_{T}$.

(13) $$\mu_{j}=\max_{i}\mu_{i}\left\{\begin{array}{ccc} \geq \mu_{T} & \Rightarrow & H_{j}\\ <\mu_{T} & \Rightarrow & H_{0} \end{array}\right.$$

where $H_{j(j>0)}$ means that the *i*th state has been identified. $H_{0}$ means that the current state cannot be identified. The threshold $\mu_{T}$ must be above 50% to prevent that the activation probabilities of two different modes exceed the threshold at the same time (as the sum of the activation probabilities is 100%). The value of the threshold can empirically be selected strictly between 50% and 100% by doing a compromise between time to detection and robustness to false detection. A low value makes the fault detection faster, while a higher value increases the robustness to false positives.

In this study, $\mu_{T}$ was empirically set to 95%.

### 2.3. Transition Probabilities

The transition matrix $\pi_{ij}$ indicates the probability for switching from a previous mode *i* to another mode *j*. These transition probabilities are implemented in the first IMM step to mix the previous estimates. For each line, the sum of the terms is equal to 1. The first line represents the transition from the nominal mode to another, and the diagonal terms indicate the probability that the system remains in the same mode between two algorithm iterations.

The terms of this matrix can be chosen to reach the best compromise between a rapid time to detection and a good robustness to false positive. Two extreme example can be highlighted:

- If $\pi_{ij}$ is set equal to the identity matrix, we show easily from Equations (4) and (5) that the mixed probabilities and the mixed estimates remains equal to the previously estimated ones. This results in skipping the first step of the IMM algorithm. In this case, the IMM behaves exactly like a standard multiple model approach, with the risk of late detection explained the introduction.
- If each term is set equal to 1/*n* (where *n* is the number of modes), then, in Step 1, the mixed estimates for all modes becomes equal to the previous overall estimate, and the mixed probabilities of activation becomes equal to 1/*n*. In this case, the previous iterations are totally forgotten, and the fault detection only relies on the current iteration. Thus, increasing the risk of false detection.

The transition matrix is generally empirically tuned between these two extreme cases. The algorithm becomes more robust to false positive if the transition matrix is close to the identity matrix. The fault detection can be made faster by increasing the non-diagonal terms. For the experimental part of this study, we adopted the following transition matrix:

$$\left(\pi_{ij}\right)=(\begin{array}{ccccc} 0.5 & \frac{0.5}{n-1} & \cdots & \cdots & \frac{0.5}{n-1}\\ 0.1 & 0.9 & 0 & \cdots & 0\\ \vdots & 0 & \ddots & \ddots & \vdots \\ \vdots & \vdots & \ddots & \ddots & 0\\ 0.1 & 0 & \cdots & 0 & 0.9 \end{array})$$

### 2.4. Immunization to Faults

To make the modes insensitive to a defined fault, the corresponding model has to be modified, compared to the nominal model.

For sensor fault, different immunization methods have been proposed:

- For a total default, the easiest way is to cancel the corresponding line in the measurement matrix *H*.
- For a partial default, the corresponding parameter can be increased in the measurement noise covariance matrix *R*.

**Figure 1:**
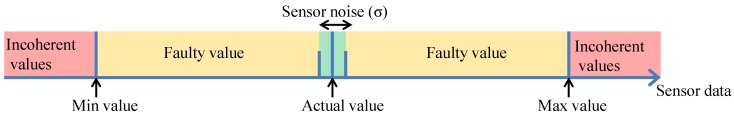
Sensor fault range.

We opted for the second solution to detect partial faults. Figure 1 displays the range of the sensor data. In the nominal state, the sensor data is close to the actual measured value; the error variance can be set according to the nominal sensor noise standard deviation σ: Var${}_{\mathrm{NOM}}=\sigma^{2}$.

To set the variance Var${}_{\mathrm{fault}}$ for the immunized mode, we have to define minimal and maximal values, above which the measurement are incoherent and can be easily rejected. For instance, the steering angle is not supposed to exceed the maximal actuator amplitude. For the acceleration, the typical maximal value is ±15 m/s${}^{2}$.

The faulty mode standard deviation $\sigma_{\max}$ is tuned to cover the whole coherent range. We get: Var${}_{\mathrm{fault}}=\sigma_{\max}^{2}$.

A similar method has been adopted for the actuator faults. The parameter of the model noise covariance *Q* is increased for the line of the evolution function which is affected by the corresponding actuator.

## 3. Probabilistic Vehicle State Observer

As explained in the previous section, the IMM algorithm compares the likelihood of different state estimations to identify system faults. The computation of the likelihoods requires an estimation of both the system state and the error on the prediction. This is typically provided by the probabilistic state observers such as the Kalman filters family.

The Kalman filter is a well known method for the state estimation of a linear system. It is based on a linear evolution model of the system, and provides both the estimate of the state vector and the covariance information about the error of this estimate.

This filter is considered recursive, as it only takes into account the previous state estimate and the current measurement.

It consists of a two-step process. In the *prediction* step, the previous state estimate is updated according to the evolution function. The linearity properties of the evolution function enable to directly compute the predicted error covariance from the previous one.

Then, the *update* step corrects the predicted state according to the current measurement. The correction gain depends on the relation between the estimation and the measurement error covariance.

For non-linear system, however, the evolution function cannot be applied to the covariance directly. To cope with non-linear system, different variants have been proposed, such as the extended Kalman filter [32], the unscented Kalman filter [33] and the divided differences filters [34]. This section introduces these filters.

### 3.1. The Extended Kalman Filter

To cope with non-linearity of the evolution function, the EKF linearises the function in the vicinity of the current state. The derivation, based on the first order Taylor development, involves the computation of the partial derivatives matrix (the Jacobian) of the evolution function. It consists of a two step process.

1. During the *prediction* step, the predicted state estimate $\hat{x}\left(k+1|k\right)$ and its predicted covariance P(k+1|k) are estimated following the non-linear evolution function *f* and its Jacobian matrix *F*, according to Equations (14) and (15).
  (14) $${\hat{x}}_{k+1|k}=f\left({\hat{x}}_{k},u_{k}\right)$$
  (15) $$P_{k+1|k}=FP_{k}F^{t}+Q$$
2. In the *update* stage, the predicted estimate is corrected according to the updated output vector $y_{k+1}$. The residual ν and its covariance Λ are evaluated according to the measurement matrix *H*.
  (16) $$\nu_{k+1}=y_{k+1}-H{\hat{x}}_{k+1|k}$$
  (17) $$\Lambda_{k+1}=HP_{k+1|k}H^{T}+R$$
  The filter gain *K* can then be calculated according to Equation (18).
  (18) $$K_{k+1}=P_{k+1|k}H^{T}\Lambda_{k+1}^{-1}$$
  Finally, the updated state estimate ${\hat{x}}_{k}$ and the updated covariance $P_{k}$ can be computed following Equations (19) and (20).
  (19) $${\hat{x}}_{k+1}={\hat{x}}_{k+1|k}+K_{k+1}\nu_{k+1}$$
  (20) $$P_{k+1}=P_{k+1|k}-K\Lambda K_{k+1}^{T}$$

*Q* and *R* are, respectively, the model and the measurement noise covariance matrices.

### 3.2. The First-Order Divided Differences Filter

The DD1 filter locally linearises the system and measurement dynamics via first-order divided differences, rather than the first order Taylor development used in the EKF. The computation of the Jacobian matrices is also no longer needed.

First, we have to define the Cholesky (or square root) decomposition of the predicted state covariance $P_{k+1|k}$, the updated state covariance $P_{k}$, the process noise covariance *Q* and the measurement noise covariance *R*:

(21) $$\begin{array}{ccccccc} Q & = & S_{v}S_{v}^{T} & \; & R & = & S_{w}S_{w}^{T}\\ P_{k+1|k} & = & S_{x_{k+1|k}}S_{x_{k+1|k}}^{T} & \; & P_{k} & = & S_{x_{k}}S_{x_{k}}^{T} \end{array}$$

The first-order divided differences matrices are defined as:

(22) $$\begin{array}{l} S_{x\hat{x}}(i,j)=\frac{f_{i}({\hat{x}}_{k}+\xi S_{x_{k},j},v_{k})-f_{i}({\hat{x}}_{k}+\xi S_{x_{k},j},v_{k})}{2\xi }\\ S_{xv}(i,j)=\frac{f_{i}({\hat{x}}_{k},v_{k}+\xi S_{v,j})-f_{i}({\hat{x}}_{k},v_{k}-\xi S_{v,j})}{2\xi }\\ S_{y\hat{x}}(i,j)=\frac{g_{i}({\hat{x}}_{k}+\xi S_{x_{k+1|k},j},n_{k})-g_{i}({\hat{x}}_{k}-\xi S_{x_{k+1|k},j},n_{k})}{2\xi }\\ S_{yv}(i,j)=\frac{g_{i}({\hat{x}}_{k},n_{k}+\xi S_{v,j})-g_{i}({\hat{x}}_{k},n_{k}-\xi S_{v,j})}{2\xi } \end{array}$$

where ξ is the disrupting parameter.

The two algorithm steps become:

1. In the *prediction* step, the predicted state vector ${\hat{x}}_{k+1|k}$ and its covariance $P_{k+1|k}$ are computed according to:
  (23) $${\hat{x}}_{k+1|k}=f\left({\hat{x}}_{k},u_{k}\right)$$
  (24) $$P_{k+1|k}=\left(S_{x\hat{x}}\right){\left(S_{x\hat{x}}\right)}^{T}+\left(S_{xv}\right){\left(S_{xv}\right)}^{T}$$
  The state covariance prediction can be factored using the square root decomposition $P_{k+1|k}=S_{x_{k+1|k}}S_{x_{k+1|k}}^{T}$ to yield:
  (25) $$S_{x_{k+1|k}}=H\left(\left[\begin{array}{cc} S_{x\hat{x}} & S_{xv} \end{array}\right]\right)$$
  where $H\left(.\right)$ is the Householder transformation.
2. In the *update* step, the predicted state will be corrected according to the new measured data *y*. The residual $\nu_{k+1}$ and its predicted covariance $\Lambda_{k+1}$ are defined by:
  (26) $$\nu_{k+1}=y_{k+1}-H{\hat{x}}_{k+1|k}$$
  (27) $$\Lambda_{k+1}=\left(S_{y\hat{x}}\right){\left(S_{y\hat{x}}\right)}^{T}+\left(S_{yv}\right){\left(S_{yv}\right)}^{T}$$
  Λ can be decomposed into $\Lambda_{k+1}=S_{y}S_{y}^{T}$ to yield:
  (28) $$S_{y}=H\left(\left[\begin{array}{cc} S_{y\hat{x}} & S_{yv} \end{array}\right]\right)$$
  The DD1 gain matrix *K* which minimizes the trace of $P_{k+1}$ is defined by:
  (29) $$K_{k+1}=S_{x_{k+1|k}}S_{y\hat{x}}\Lambda_{k+1}^{-1}$$
  Finally, the updated state vector ${\hat{x}}_{k+1}$ and its error covariance matrix $P_{k+1}$ are calculated with:
  (30) $${\hat{x}}_{k+1}={\hat{x}}_{k+1|k}+K_{k+1}\nu_{k+1}$$
  (31) $$P_{k+1}=P_{k+1|k}-K_{k+1}\Lambda_{k+1}K_{k+1}^{T}$$
  Here, again, the error covariance matrix can be decomposed into $P_{k+1}={\hat{S}}_{x}{\hat{S}}_{x}^{T}$ with:
  (32) $$S_{x_{k+1}}=H\left(\left[\begin{array}{cc} S_{x_{k+1|k}}-K_{k+1}S_{y\hat{x}}\; & K_{k+1}S_{yv} \end{array}\right]\right)$$

### 3.3. The Unscented Kalman Filter

The UKF is a derivative-free filter based on a constellation of 2n+1 points (the sigma points) in the vicinity of the current state vector (where *n* is the length of the state vector).

Instead of derivating the non-linear evolution function, the predicted covariance is estimated according to the weighted distribution of the predicted evolution of the sigma points.

The first step consists in calculating the sigma points $X_{k-1}$ and the corresponding weights:

(33) $$X_{k}=\left[{\hat{x}}_{k}\;{\hat{x}}_{k}+\gamma \sqrt{P_{k}}\;{\hat{x}}_{k}-\gamma \sqrt{P_{k}}\right]$$

where $\sqrt{P_{k}}$ is the Cholesky decomposition of $P_{k}$.

The weights $W_{i}^{\left(m\right)}$ and $W_{i}^{\left(c\right)}$ are defined according to the parameters κ and η:

(34) $$\begin{array}{cc} W_{0}^{\left(m\right)}=\frac{\kappa }{\eta +\lambda } & W_{0}^{\left(c\right)}=\frac{\kappa }{\eta +\lambda }+1-\alpha^{2}+\beta \\ W_{i}^{\left(m\right)}=W_{i}^{\left(c\right)}=\frac{1}{2(\eta +\lambda )}\; & \forall i\in [1,2n] \end{array}$$

1. In the *prediction* step, the predicted state vector ${\hat{x}}_{k+1|k}$ and its covariance $P_{k+1|k}$ are computed according to:
  (35) $$X_{k+1|k}^{*}=f\left(X_{k},u_{k}\right)$$
  The estimated state is the weighted centre of the sigma points.
  (36) $${\hat{x}}_{k+1|k}=\sum_{i=0}^{2n}W_{i}^{\left(m\right)}X_{i,k+1|k}^{*}$$
  The estimated covariance is estimated from the distribution of the sigma points:
  (37) $$P_{k+1|k}=\sum_{i=0}^{2n}W_{i}^{\left(c\right)}\left[X_{i}^{*}-{\hat{x}}_{k+1|k}\right]{\left[X_{i}^{*}-{\hat{x}}_{k+1|k}\right]}^{T}+Q$$
2. In the *update* step, new sigma points $X_{k+1|k}$ are drawn with the estimated covariance to estimate the predicted measurement vector ${\hat{y}}_{k}$.
  (38) $$X_{k+1|k}=\left[{\hat{x}}_{k+1|k}\;{\hat{x}}_{k+1|k}\pm \gamma \sqrt{P_{k+1|k}}\right]$$
  (39) $$Y_{k+1}=HX_{k+1|k}$$
  (40) $${\hat{y}}_{k+1}=\sum_{i=0}^{2n}W_{i}^{\left(m\right)}Y_{i,k+1}$$
  The residual covariance Λ is estimated by:
  (41) $$\Lambda_{k+1}=\sum_{i=0}^{2n}W_{i}^{\left(c\right)}\left[Y_{i,k+1}-{\hat{y}}_{k+1}\right]{\left[Y_{i,k+1}-{\hat{y}}_{k+1}\right]}^{T}+R$$
  The filter gain *K* is then computed with:
  (42) $$K_{k+1}=P_{\hat{x}{\hat{y}}_{k+1}}\Lambda_{k+1}^{-1}$$
  where
  (43) $$P_{\hat{x}{\hat{y}}_{k+1}}=\sum_{i=0}^{2n}W_{i}^{\left(c\right)}\left[X_{i,k+1|k}-{\hat{x}}_{k+1|k}\right]{\left[Y_{i,k+1}-{\hat{y}}_{k+1}\right]}^{T}$$
  Finally, the state estimate and its covariance are updated according to the gain and the current measurement vector *y*:
  (44) $${\hat{x}}_{k+1}={\hat{x}}_{k+1|k}+K_{k+1}\left(y_{k+1}-{\hat{y}}_{k+1}\right)$$
  (45) $$P_{k+1}=P_{k+1|k}-K_{k+1}\Lambda_{k+1}^{-1}K_{k+1}^{T}$$

## 4. Vehicle Dynamics Model

Model-based FDI schemes for SBW systems generally rely on a simplified linear single-track model, generally called the bicycle model (see [5,35,36]). However, BBW systems generates asymmetrical braking forces, which cannot be treated with the bicycle model. Moreover, this linear model does not cope with the non-linearities due to large tyre slips, which are likely to occur in case of braking failure.

Thus, we have opted for simplified non-linear two-track model, which is presented in this section.

### 4.1. Two-Track Vehicle Model

**Figure 2:**
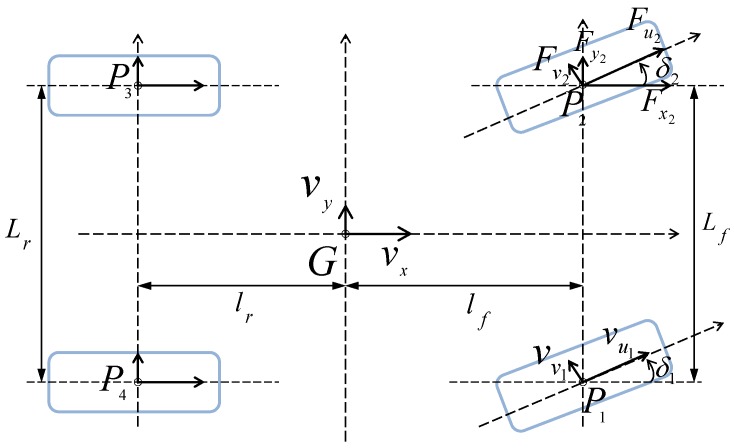
The two-track model.

Based on Newton’s second law of motion (see Equation (46), the longitudinal, lateral and yaw accelerations $a_{x}$, $a_{y}$, and $\ddot{\psi }$ at the centre of gravity *G* are expressed according to the estimated tyre–road forces $F_{x_{i}}$, where i∈[1,2,3,4] indicates the wheel index, as represented in Figure 2. $I_{z}$ is the vehicle moment of inertia, $F_{ext}$ represents the external forces acting on the vehicle (rolling and air resistance), supposed longitudinal and steady.

(46) $$\left\{\begin{array}{ccc} a_{x} & = & \frac{1}{M}\left(F_{ext}+\sum_{i=1}^{4}F_{x_{i}}\right)\\ a_{y} & = & \frac{1}{M}\sum_{i=1}^{4}F_{y_{i}}\\ \ddot{\psi } & = & \frac{1}{I_{z}}\sum_{i=1}^{4}∥\vec{GP_{i}}\wedge \vec{F_{i}}∥ \end{array}\right.$$

The evolution of the vehicle longitudinal and lateral velocities $v_{x}$ and $v_{y}$ at point *G* are then expressed according to Equation (47).

(47) $$\left\{\begin{array}{ccc} {\dot{v}}_{x} & = & a_{x}+v_{y}\dot{\psi }\\ {\dot{v}}_{y} & = & a_{y}-v_{x}\dot{\psi } \end{array}\right.$$

### 4.2. Tyre–Road Force Estimation

Several models have been presented in the literature to estimate the tyre–road forces, according to the tyre longitudinal and lateral tyre slip rates $\lambda_{i}$ and $\alpha_{i}$ defined by Equation (48).

(48) $$\left\{\begin{array}{ccc} \lambda_{i} & = & \frac{R_{i}\omega_{i}-v_{u_{i}}}{\max(R_{i}\omega_{i},v_{u_{i}})}\\ \alpha_{i} & = & \delta_{i}-\arctan\left(\frac{v_{v_{i}}}{v_{u_{i}}}\right) \end{array}\right.$$

where $v_{u_{i}}$ and $v_{v_{i}}$ are the expression of the wheel velocity $\vec{v_{i}}$ along the wheel main axis. This velocity is computed from the vehicle velocity at point *G* and its turn speed vector $\vec{\Omega }$.

(49) $$\vec{v_{i}}=\vec{v_{G}}+\vec{\Omega }\wedge \vec{GP_{i}}$$

where $P_{i}$ is the position of the centre of the wheel.

The model proposed by Dugoff et al. [37] has the advantages of relying on only two parameters, the longitudinal and lateral tyre stiffness $C_{x}$ and $C_{y}$, which can be easily estimated with experimental tests.

The road–tyre force, which presents a saturation for high values of $\lambda_{i}$ and $\alpha_{i}$, is expressed in the wheel main axis by:

(50) $$\left\{\begin{array}{ccc} F_{u_{i}} & = & C_{x}\frac{\lambda_{i}}{1-\lambda_{i}}k_{i}\\ F_{v_{i}} & = & C_{y}\frac{\tan\alpha_{i}}{1-\lambda_{i}}k_{i} \end{array}\right.$$

with

(51) $$k_{i}=\left\{\begin{array}{ccc} \left(2-\sigma_{i}\right)\sigma_{i} & if & \sigma_{i}<1\\ 1 & if & \sigma_{i}\geq 1 \end{array}\right.$$

and

(52) $$\sigma_{i}=\frac{\left(1-\lambda_{i}\right)\mu_{i}F_{z_{i}}}{2\sqrt{C_{x}^{2}\lambda_{i}^{2}+C_{y}^{2}\tan^{2}\alpha_{i}}}$$

The repartition of the normal forces $F_{z_{i}}$ on the wheels is given by:

(53) $$\left\{\begin{array}{c} F_{z_{1}}=\frac{M}{l_{f}+l_{r}}\left[l_{r}g-ha_{x}\left(\frac{1}{2}+\frac{ha_{y}}{L_{f}g}\right)\right]\\ F_{z_{2}}=\frac{M}{l_{f}+l_{r}}\left[l_{r}g-ha_{x}\left(\frac{1}{2}-\frac{ha_{y}}{L_{f}g}\right)\right]\\ F_{z_{3}}=\frac{M}{l_{f}+l_{r}}\left[l_{f}g+ha_{x}\left(\frac{1}{2}-\frac{ha_{y}}{L_{r}g}\right)\right]\\ F_{z_{4}}=\frac{M}{l_{f}+l_{r}}\left[l_{f}g+ha_{x}\left(\frac{1}{2}+\frac{ha_{y}}{L_{r}g}\right)\right] \end{array}\right.$$

where $l_{f}$ (respectively, $l_{r}$) is the distance between *G* and the front (respectively, rear) axle, *h* is the height of the centre of gravity, and $L_{f}$ and $L_{r}$ the front and rear axle widths. Finally, the forces are expressed in the vehicle referential according to the steering angle $\delta_{i}$.

(54) $$\left\{\begin{array}{ccc} F_{x_{i}} & = & F_{u_{i}}\cos\delta_{i}-F_{v_{i}}\sin\delta_{i}\\ F_{y_{i}} & = & F_{v_{i}}\cos\delta_{i}+F_{u_{i}}\sin\delta_{i} \end{array}\right.$$

### 4.3. Actuators Models

To detect actuator faults, it is mandatory to take into account the actuators in the model. In our case, the actuators are the brake callipers, and the motor on the steering rack to settle the front steering angle.

The steering motor is directly controlled by angle. Its response is very fast compared to the vehicle dynamics, so that the actuation delay can be neglected. The actual angle $\delta_{i}$ is then supposed equal to the commanded angle $\delta_{u}$.

(55) $$\delta_{i}=\delta_{u}$$

Concerning the brakes, the evolution of the wheel dynamics requires the estimation of the braking torque which is generally not measurable.

In the case of an electromagnetic braking (for instance, for regenerative braking), which are standard devices on electric vehicles, the torque can be estimated from the voltage and the current applied to the solenoid.

In the case of conventional hydraulic brakes, the braking torque in controlled according to the hydraulic pressure on the calliper. This braking pressure is typically measured by the ESC control unit and could be used to estimate the braking torque.

In our test vehicle (see Section 5), which was originally not fitted with an ESC system, a hydraulic unit was integrated into the braking circuit to maintain a commanded slip rate. However, the braking pressure cannot be measured and, therefore, the estimation of the actual braking torque is more difficult. The following model, detailed in [38], was consequently implemented.

The ESC control unit contains 12 solenoid valves and a pump. According to the combination of activated valves, the hydraulic pressure at the callipers can be controlled in the following four different modes:

- “normal” mode: The pressure from the master cylinder is normally transmitted to the calliper.
- “maintained” mode: The calliper pressure is maintained constant.
- “released” mode: The pump is activated to release the pressure.
- “braked” mode: The pressure is increased, without any driver action.

In a first step, the resulting braking torque $T_{b_{i}}$ is supposed to vary linearly according to the state (see Equation (56). The parameters $\Delta T_{b}$ and $\Delta T_{r}$ are set experimentally. Of course, the braking torque cannot be negative.

(56) $$T_{b_{i}}\left(t+\Delta t\right)=\left\{\begin{array}{ccc} T_{b_{i}}\left(t\right)+\Delta T_{b} & \mathrm{if} & \;\mathrm{state}\;\mathrm{is}\;“\mathrm{braked}”\\ T_{b_{i}}\left(t\right) & \mathrm{if} & \mathrm{state}\;\mathrm{is}\;“\mathrm{maintained}”\\ T_{b_{i}}\left(t\right)-\Delta T_{r} & \mathrm{if} & \;\mathrm{state}\;\mathrm{is}\;“\mathrm{released}” \end{array}\right.$$

Finally, the wheel turn acceleration is computed from the estimated braking torque, the tyre–road longitudinal force and the effective wheel radius $R_{i}$:

(57) $${\ddot{\omega }}_{i}=\frac{1}{I_{w_{i}}}\left(T_{b_{i}}-R_{i}F_{u_{i}}\right)$$

### 4.4. Implementation of the Probabilistic Observers

The output vector is composed of the different sensor output variables: $y=[a_{x},a_{y},\dot{\psi },\omega_{1},\omega_{2},$$\omega_{3},\omega_{4},\delta_{i}{]}^{t}$.

For a vehicle dynamics observer, the state vector $\hat{x}$ is composed of the estimates of the velocities ${\hat{v}}_{x}$ and ${\hat{v}}_{y}$. To allow the sensor fault detection, $\hat{x}$ has been augmented to obtain an estimate of each sensor data. Then, we obtain: $\hat{x}={\left[{\hat{v}}_{x},{\hat{v}}_{y},{\hat{a}}_{x},{\hat{a}}_{y},\hat{\dot{\psi }},{\hat{\omega }}_{1},{\hat{\omega }}_{2},{\hat{\omega }}_{3},{\hat{\omega }}_{4},{\hat{\delta }}_{i}\right]}^{t}$.

According to the previously mentioned vehicle model (see Equations (46), (47), (55) and (57), the following evolution function *f* is adopted for the observers implementation:

(58) $$f=\left\{\begin{array}{ccc} {\hat{v}}_{x}\left(k+1\right) & = & {\hat{v}}_{x}\left(k\right)+\left({\hat{a}}_{x}\left(k\right)+{\hat{v}}_{y}\left(k\right)\hat{\dot{\psi }}\left(k\right)\right)\Delta t\\ {\hat{v}}_{y}\left(k+1\right) & = & {\hat{v}}_{y}\left(k\right)+\left({\hat{a}}_{y}\left(k\right)-{\hat{v}}_{x}\left(k\right)\hat{\dot{\psi }}\left(k\right)\right)\Delta t\\ {\hat{a}}_{x}\left(k+1\right) & = & \frac{1}{M}\left(F_{ext}+\sum_{i=1}^{4}F_{x_{i}}\right)\\ {\hat{a}}_{y}\left(k+1\right) & = & \frac{1}{M}\sum_{i=1}^{4}F_{y_{i}}\\ \hat{\dot{\psi }}\left(k+1\right) & = & \hat{\dot{\psi }}\left(k\right)+\ddot{\psi }\left(k\right)\Delta t\\ {\hat{\omega }}_{1}\left(k+1\right) & = & {\hat{\omega }}_{1}\left(k+1\right)+\frac{\Delta t}{I_{w_{1}}}\left(T_{b_{1}}-R_{1}F_{u_{1}}\right)\\ {\hat{\omega }}_{2}\left(k+1\right) & = & {\hat{\omega }}_{2}\left(k+1\right)+\frac{\Delta t}{I_{w_{2}}}\left(T_{b_{2}}-R_{2}F_{u_{2}}\right)\\ {\hat{\omega }}_{3}\left(k+1\right) & = & {\hat{\omega }}_{3}\left(k+1\right)+\frac{\Delta t}{I_{w_{3}}}\left(T_{b_{3}}-R_{3}F_{u_{3}}\right)\\ {\hat{\omega }}_{4}\left(k+1\right) & = & {\hat{\omega }}_{4}\left(k+1\right)+\frac{\Delta t}{I_{w_{4}}}\left(T_{b_{4}}-R_{4}F_{u_{4}}\right)\\ \hat{\delta }\left(k+1\right) & = & \delta_{u}\left(k+1\right) \end{array}\right.$$

The same evolution model (Equation (58)) is implemented for each of the 14 considered modes. The only differences between the different observers are the parameters of the noise matrices: measurement noise matrix *R* for the immunization to sensor faults, and model noise matrix *Q* for the actuator faults, as explained in Section 2.4.

## 5. Experimental Validation

The FDI algorithm was validated with experimental data, recorded with an experimental test vehicle on the Satory test tracks, in Versailles (France). The test scenarios included standards driving situations (normal urban driving) and critical maneuvers (emergency braking).

### 5.1. Experimental Vehicle

The test prototype was a SECMA f16, a leisure convertible rear-wheel drive vehicle. This prototype was modified to integrate drive-by-wire functionalities (SBW and BBW).

The vehicle has been fitted with proprioceptive sensors:

- an inertial measurement unit (IMU) with three-axis accelerometer and gyrometer (type Xsense MTI-G);
- four wheel turn rate sensors;
- a front wheel steering angle sensor; and
- a steering wheel angle sensor.

Thanks to an ESC hydraulic control unit, integrated on the braking hydraulic circuit between the master cylinder and the callipers, the braking pressure can be adapted to maintain a commanded slip rate for each wheel. More information on this system can be found in [38].

The sensor acquisition and the actuator control is ensured by a programmable electronic control unit (ECU, type AFT PROtronic), which integrates a micro-controller, standard interfaces (PWM, A/D converters) and CAN interfaces for communication between PC and data logging. The data logging sampling frequency is limited by the CAN interface features (max: 100 Hz).

### 5.2. Comparison of the Probabilistic Observers

A fast and reliable fault detection via a model-based FDI algorithm requires an accurate observation of the vehicle state. Moreover, to avoid false detection, the observation has to remain consistent, i.e., that the predicted error covariance stays compatible with the actual estimation error.

On another side, a major concern in the Multiple Model approaches is the computational load. Indeed, the probabilistic filters are computed for each model in parallel, thus multiplying the operation numbers. The observer has to achieve a reasonable computational time to stay compatible with an embedded real time architecture.

In a first step, we selected among the different considered observers (EKF, UKF and DD1) the one which suits the most the IMM approach, regarding the accuracy and the consistency of the observation and the computational time efficiency.

#### 5.2.1. Observation Accuracy

The estimated state is composed of the longitudinal and the lateral speeds, and the estimates of the eight proprioceptive sensors (X- and Y-accelerations, yaw rate, wheel speeds and steering angle). To check the observers accuracy, we compared the estimates to reference sensor signals during highly dynamical driving situations like an emergency braking and sharp turns.

**Figure 3:**
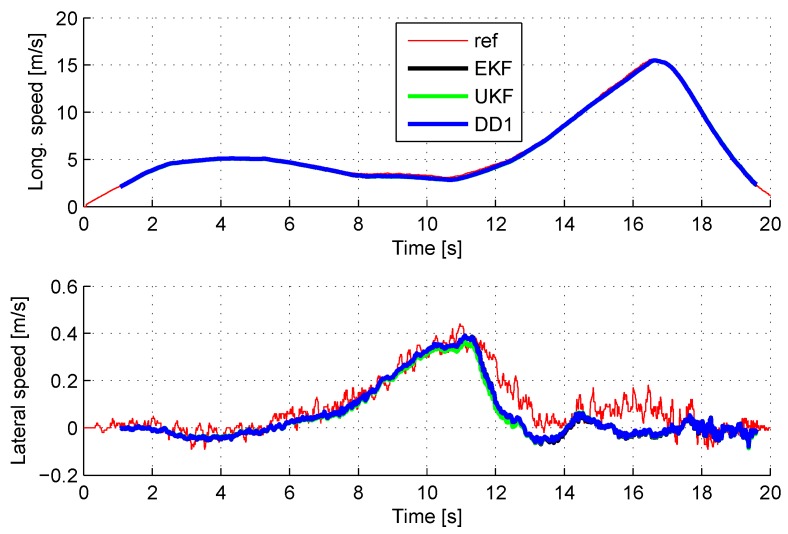
Longitudinal and lateral speed estimates during an emergency braking.

Figure 3 compares the estimation of the longitudinal speed during an emergency braking maneuver, with the three considered observers. The reference speed is given by a Correvit sensor (KISTLER S-350). The estimates given by the EKF, UKF and DD1 observers are, respectively, plotted in black, green and blue.

The driving maneuver consisted of a slight acceleration (from 1 s to 8 s), followed by a sharp right turn (from 8 s to 11 s), then a strong acceleration (from 11 s to 17 s), and finally an emergency braking (from 17 s to 20 s), with activation of the ABS system on the front wheels during the braking.

The three observers give a very good estimation of the vehicle velocity; the difference with the reference sensor remains below 0.3 m/s, and it is difficult to draw clear distinctions between the curves. Consequently, no significant differences can be made in terms of accuracy. The same conclusion can be made for the other estimated data. Table 1 details the root mean square (RMS) of the difference between the raw sensor signal and the estimated data for the vehicle sensors during the same maneuver. The estimation error remains of the order of the sensor noises.

To sum up, EKF, UKF and DD1 provide accurate estimation of the vehicle state, even in the case of a highly dynamic driving situation.

#### 5.2.2. Observer Consistency

**Figure 4:**
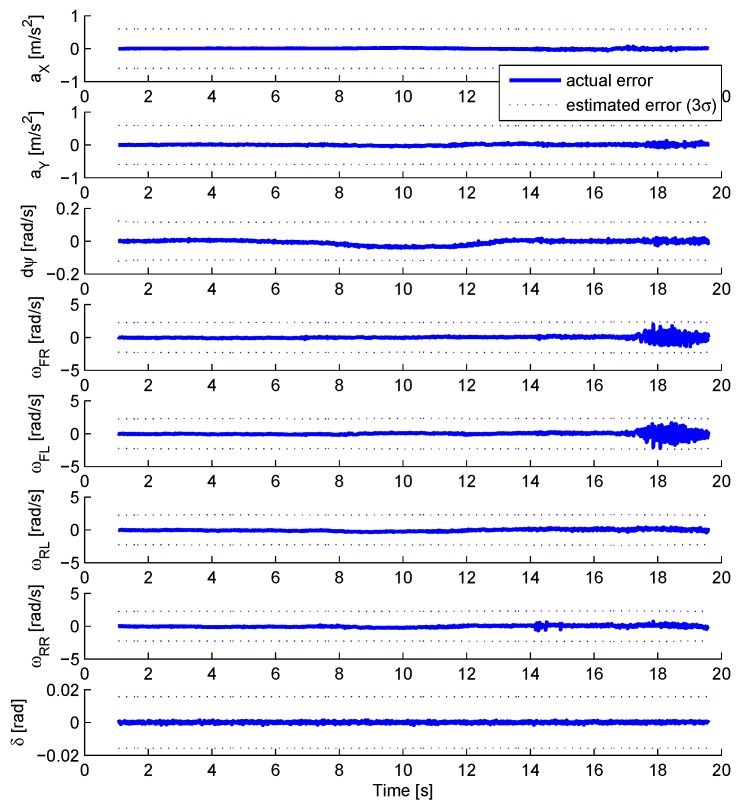
Estimation error consistency check during an emergency braking.

Figure 4 compares the estimation error for the different vehicle sensors to the estimated precision margin. The precision margin correspond to ± 3σ where the standard deviation σ is issued from the covariance matrix. Only the results for the EKF observer are displayed. The other observers present very similar results.

All signals remain inside the estimated precision margin, even during the ABS activation, observable at time 17 s on the front wheel speed signals. The observer is consistent.

#### 5.2.3. Computational Time

The computational load has been measured with the Matlab function *profile* with the same input signals as previously (see Table 2).

The UKF needs six times more resources than the EKF. This is even more spectacular with the DD1, which is 15 times more time-consuming. This can be explained by the number of call of the evolution function during each algorithm cycle. The UKF does not require the computation of the Jacobian matrix, however the evolution function, which is quite complex in our case (computation of the tyre/road forces), has to be evaluated for each of the 21 sigma points, thus increasing dramatically the computational costs. The DD1 algorithm necessitates many calls of the evolution function for the computation of the divided differences matrix, together with the required resources for the Cholesky triangulations.

#### 5.2.4. Conclusion on the Observers Comparison

This study has shown no significant differences regarding the accuracy and the consistency of the considered observers. However, in terms of computational time, the EKF is in our case much more efficient and therefore more adequate for the IMM implementation. In the following, we consequently implement the EFK for the IMM-based FDI algorithm.

### 5.3. Sensor Fault Detection Performances

The test scenario consists of an urban driving situation with a slight acceleration followed by a right turn.

For each sensor, a partial fault has been simulated by adding an offset to one of the initially measured data. The amplitude of this fault has been varied to investigate the detection of partial faults. The fault occurs at time t=4 s and disappears at time t=5 s.

**Figure 5:**
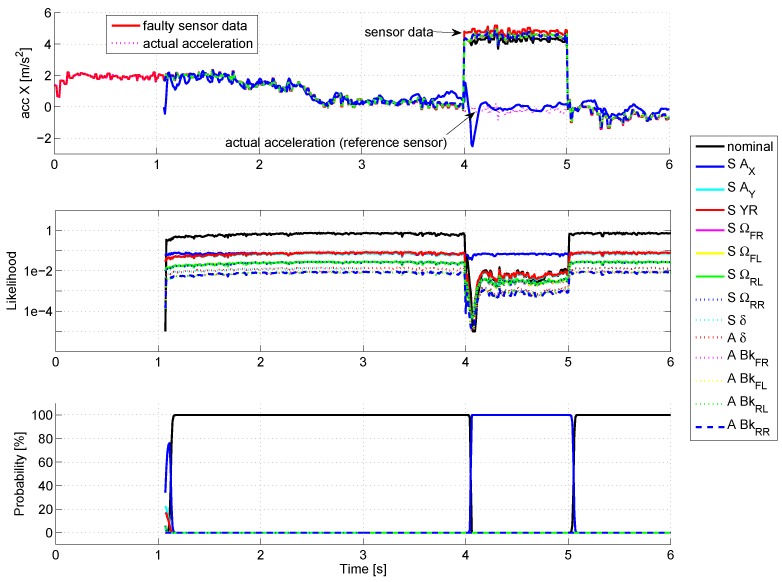
Fault on the X-acceleration sensor.

**Figure 6:**
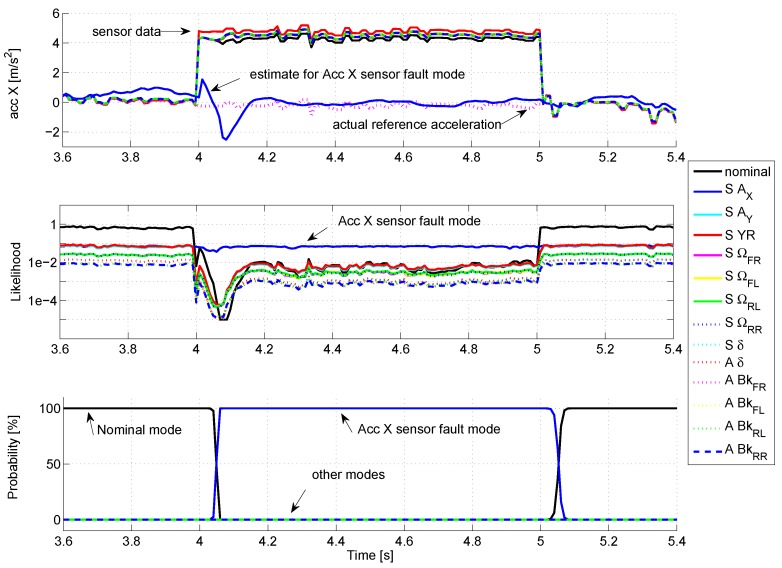
Fault on the X-acceleration sensor (zoom).

For the first simulation (see Figure 5 , zoomed in at Figure 6), a fault on the X-acceleration sensor is simulated with a 5 m/s${}^{2}$ amplitude.

The curves at the top display the estimates of X-acceleration for each mode. Before the fault occurrence, all the modes follow precisely the measured signal, except for the $SA_{X}$ mode, which considers a very large noise for this sensor, thus estimating only roughly the actual signal. By looking at the likelihood (curves in the middle, with logarithmic scale), the nominal mode present a very good likelihood (value close to 1), while for the other modes, the likelihood is quite poor (<0.1), so that the probability of activation (at the bottom) of the nominal mode stays close to 100%.

When the fault occurs (time = 4 s), the estimate of all modes (except the $SA_{X}$ mode) stick to the faulty sensor value, so that the likelihoods drop rapidly. The mode $SA_{X}$, which is almost not affected by the sensor fault, presents the best likelihood. Consequently, its probability of activation rises progressively to 100%, meaning that a fault occurred on the X-acceleration sensor: the sensor fault is thus correctly detected. The time to detection reaches 60 ms, i.e., six algorithm cycles.

Similar results are obtained with the other vehicle sensors.

To investigate the limit of the detectable faults, the same simulation was carried out with different fault amplitudes for each sensor. The detectable faults thresholds are given in Table 3.

### 5.4. Actuator Fault Detection

The detection of actuator faults is generally more complicated than for sensor faults, as it requires a precise model of the actuator operation.

Concerning the steering actuator, the steering control system is supposed to be very accurate and fast, compared to the vehicle dynamics, so that the actuator delay can be neglected, as shown in Equation (55). To simulate an actuator fault, the target steering angle $\delta_{u}$ has been modified by adding an offset value, while the actual steering angle is not modified. The scenario is the same as for the sensor faults, the fault is activated between time = 4 s and 5 s with an amplitude of 0.1 rad.

**Figure 7:**
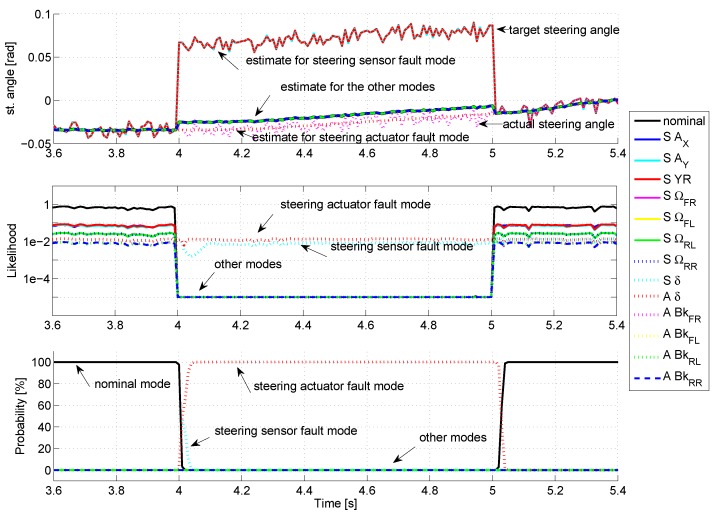
Fault on the steering actuator.

The results are displayed in Figure 7. When the fault occurs, all the modes present a large likelihood drop, except the modes Sδ and Aδ which are, respectively, insensitive to steering sensor and steering actuator faults, for which the likelihood drop is weak. For the Sδ mode, the estimated steering angle sticks to the target angle, as the measured angle is almost not taken into account, while the Aδ estimate correctly the actual angle. Its likelihood remains consequently better than the Sδ mode and the corresponding probability of activation rises to 100%. The fault detection threshold is crossed after 40 ms, confirming the correct detection and identification of the actuator fault.

Concerning the brakes, as explained in Section 4.3, as no information on the braking pressure is available for the test vehicle, the actual braking torque is very difficult to estimate. The implemented model provides only a rough prediction of the braking torque. The detection of a brake actuator requires consequently a very sensitive setting for the corresponding modes. Under this condition, it was not possible to find a satisfying compromise between fault detection and robustness to false detection.

In future work, an implementation of this algorithm on a driving simulation software is planned to investigate the detection of brake faults under more favourable conditions (e.g., availability of the braking pressure and precise knowledge of the actuator properties).

### 5.5. Robustness to False Detection

**Figure 8:**
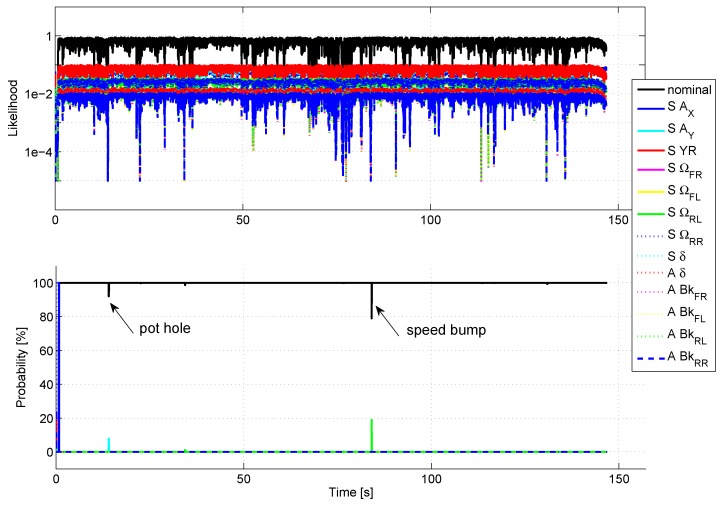
Robustness to false detection in urban driving.

To check the robustness to false detection, the sensor signals have been recorded during an urban driving scenario (150 s), including sharp turns, roundabout, emergency braking and road bumps. No false detection has been reported during this test (see Figure 8). The passing of a pothole (time = 14 s) and a speed bump (at time 84 s) provokes a slight nominal probability drop.

### 5.6. Fault Tolerant Velocity Estimation

Next to the fault detection, the IMM algorithm provides a fault-tolerant observation via the overall estimate. This can be precious when implementing active fault-tolerant control of the vehicle, which relies on the velocity of the vehicle.

**Figure 9:**
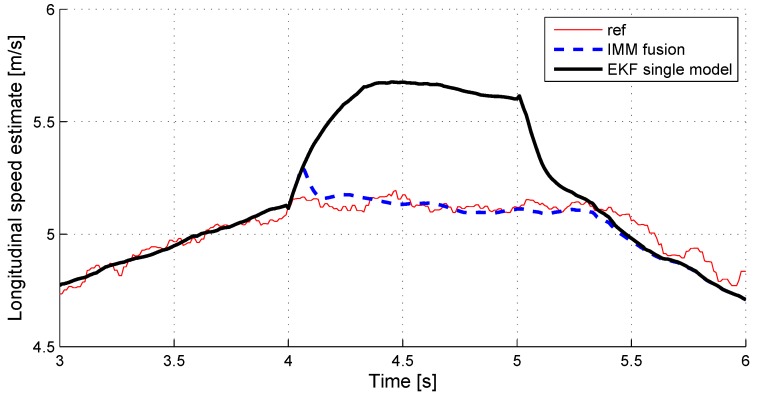
Longitudinal speed estimate with X-acceleration sensor fault.

**Figure 10:**
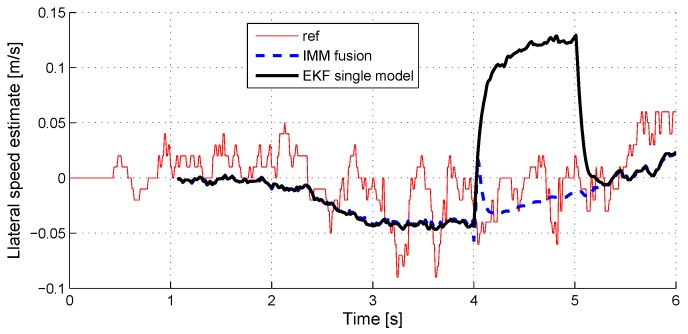
Lateral speed estimate with Y-acceleration sensor fault.

We check here the sensitivity of the velocity estimation to sensor faults. Figure 9 (respectively, Figure 10) shows the overall estimate of the longitudinal (respectively, lateral) vehicle velocity in case of a strong X-acceleration (respectively, Y-acceleration) sensor fault (+5 m/s${}^{2}$ offset). For information, the estimate given by a simple model EKF observer is displayed.

It shows that the velocity estimation remains very accurate, even during a strong sensor fault.

## 6. Conclusions

In this study, we discussed the detection and the isolation of embedded sensor and actuator faults for a drive-by wire vehicle via an adapted IMM algorithm. The compatibility of different probabilistic observers was investigated: the extended Kalman filter, the unscented Kalman filter and the first-order divided differences filter. If the three filters give similar performances, the EKF outperforms the others in terms of computational costs. The results, based on experimental data, show that total and partial faults for each of the proprioceptive sensors can be detected and identified within a short number of algorithm cycles. The detection of actuator faults seems more tedious: while a steering actuator fault can be identified correctly, the detection of braking faults has not been achieved and would require more investigations. The robustness of the detection (false detection rate) seems to be adequate, and should be confirmed with complementary experimental data.

In parallel, this approach allows a fault-tolerant estimation of the vehicle dynamics, which is interesting for vehicle control purpose. Future work will focus on the implementation of a fault-tolerant control strategy, which could take into consideration the identified faults to maintain a degraded vehicle controllability state in case of faults. 

## Figures and Tables

**Table 1:** Estimation error RMS during normal driving situation.

Sensor	$\omega_{i}$	$a_{x}$	$a_{y}$	$\dot{\psi }$	δ
EKF	0.1261	0.1199	0.1093	0.0046	0.00045
UKF	0.1215	0.1191	0.1234	0.0045	0.00046
DD1	0.1217	0.1198	0.1234	0.0045	0.00046
unit	rad/s	m/s${}^{2}$	m/s${}^{2}$	rad/s	rad

**Table 2:** Computational load of the filters.

Observer	Call of the Evolution Function per Algo Cycle	Total Computational Time
EKF	1	1.37 s
UKF	21	11.5 s
DD1	37	26.6 s

**Table 3:** Amplitude of detectable sensor faults.

Sensor	$\omega_{i}$	$a_{x}$	$a_{y}$	$\dot{\psi }$	δ
ampl.	5.0 rad/s	1.6 m/s${}^{2}$	2.7 m/s${}^{2}$	0.35 rad/s	0.055 rad

## Abbreviations

The following abbreviations are used in this manuscript:

LIVIC	Laboratory on Interactions Vehicle-Infrastructure-Driver
IBISC	Informatique, Biologie Intégrative et Systèmes Complexes
DBW	Drive-By-Wire
BBW	Brake-By-Wire
SBW	Steer-By-Wire
FDI	Fault Detection and Isolation
IMM	Interacting Multiple Models
HMI	Human Machine Interface
ESC	Electronic Stability Control
INS	Inertial Navigation Sensor
SAS	Steering wheel Angle Sensor
MM	Multiple Models
EKF	Extended Kalman Filter
DD1	First-order Divided Differences
UKF	Unscented Kalman Filter
